# Supplementary material for: Immune memory shapes human polyclonal antibody responses to H2N2 vaccination
Source: bioRxiv. 2023 Aug 24:2023.08.23.554525. Preprint. [Version 1] doi: 10.1101/2023.08.23.554525 (PMC10541104; doi:10.1101/2023.08.23.554525)
Supplement: Supplement 1 [file media-1.pdf]

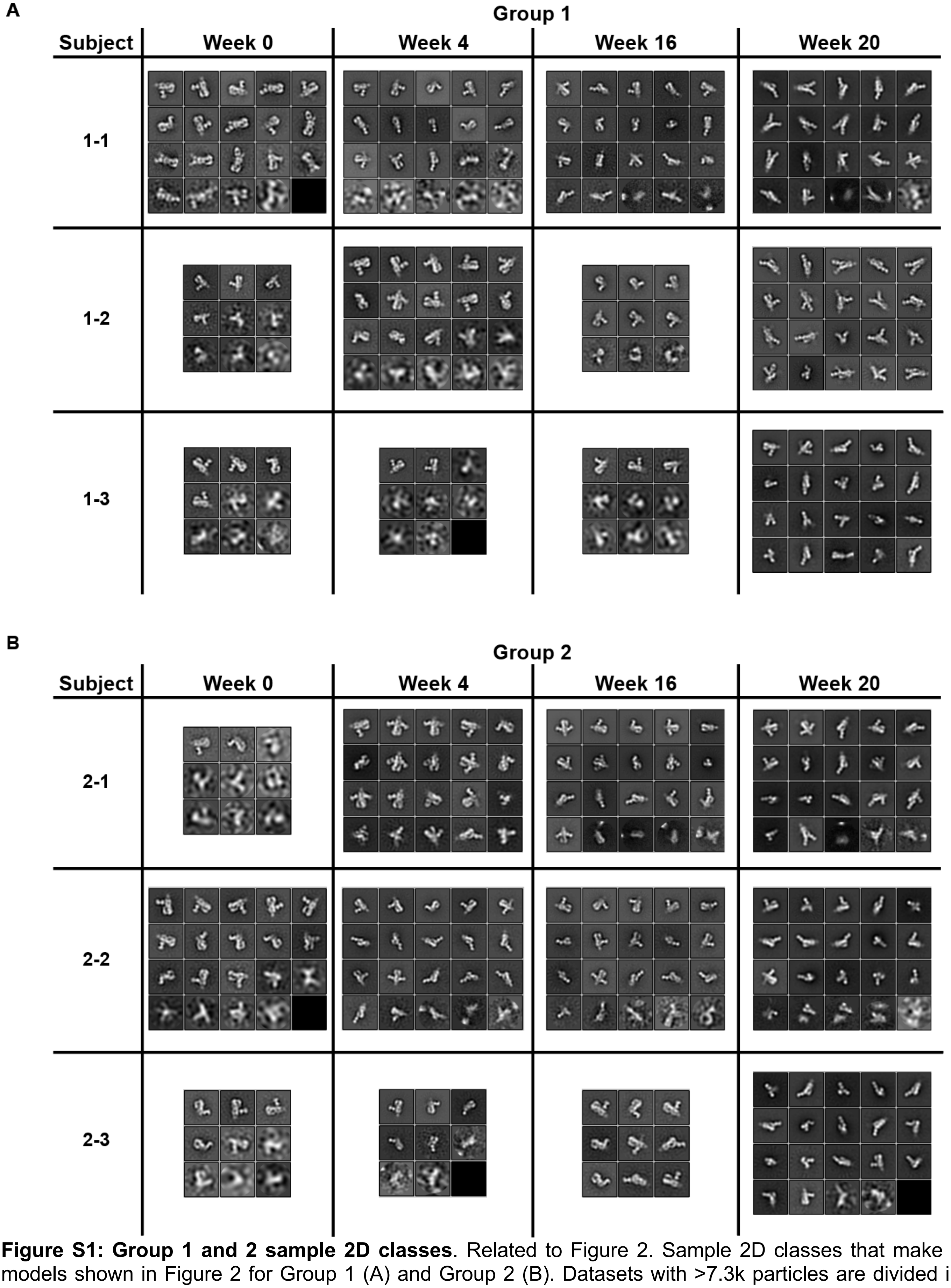

Figure S1: Group 1 and 2 sample 2D classes. Related to Figure 2. Sample 2D classes that make up 3D models shown in Figure 2 for Group 1 (A) and Group 2 (B). Datasets with >7.3k particles are divided into 20 classes while those with <7.3k particles are divided into 9. All 2D classification datasets are shown in order of descending particle count.

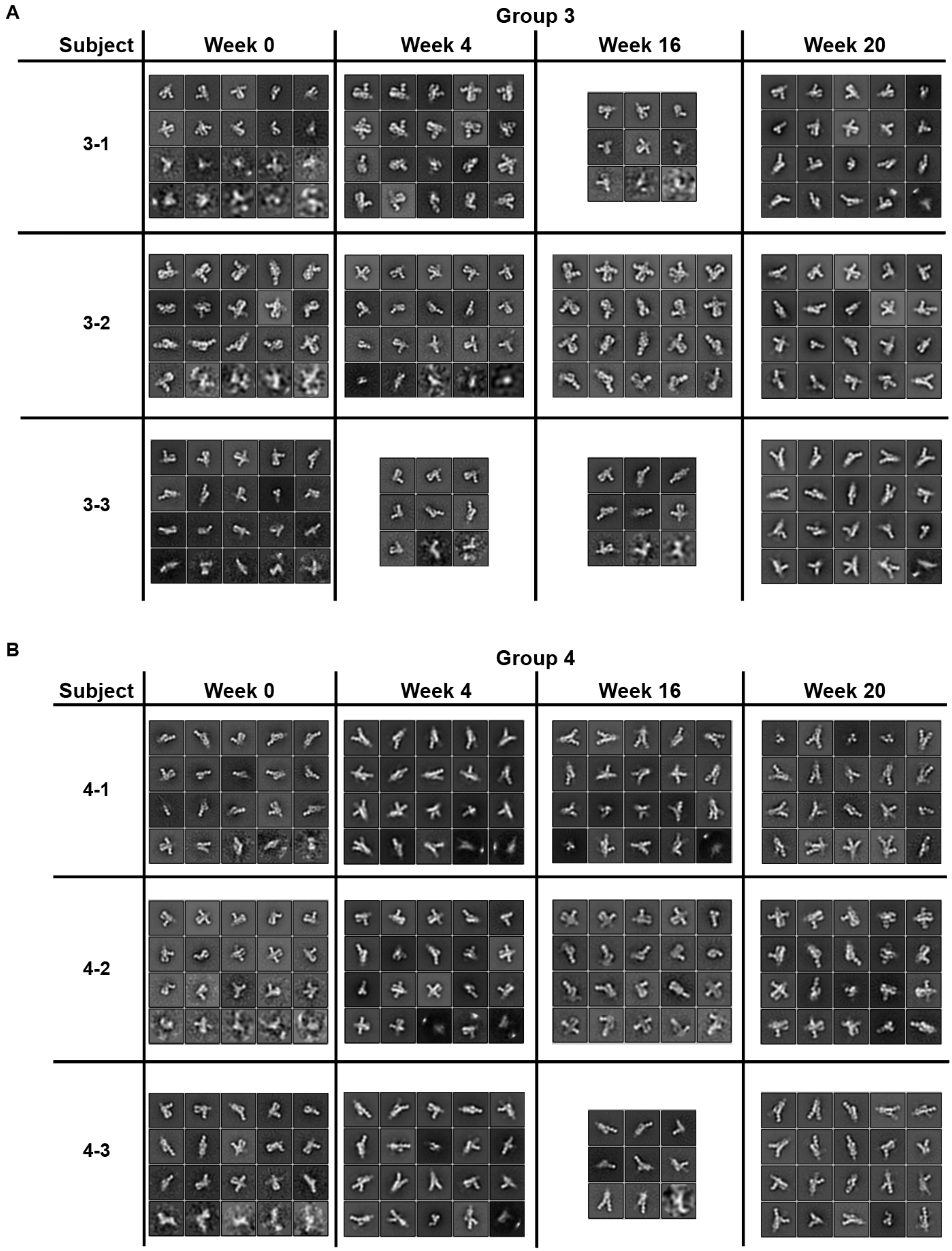

Figure S2: Group 3 and 4 sample 2D classes. Related to Figure 2. Sample 2D classes that make up 3D models shown in Figure 2 for Group 3 (A) and Group 4 (B). Datasets with >7.3k particles are divided into 20 classes while those with <7.3k particles are divided into 9. All 2D classification datasets are shown in order of descending particle count.

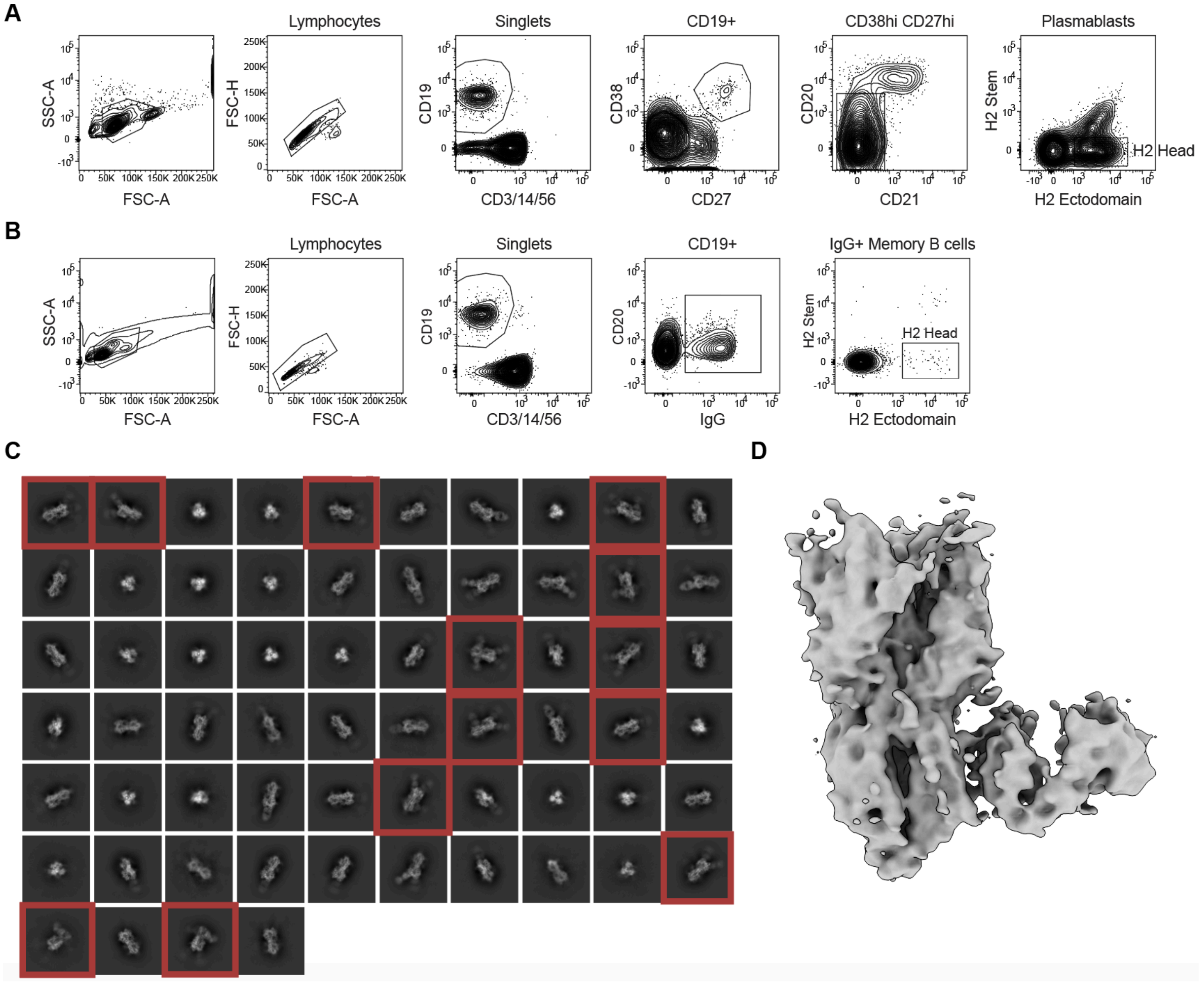

Figure S3: B-cell sorting strategy and polyclonal stem responses observed by cryo-EM. Related to Figure 4 and 5. Flow cytometry gates used to detect and sort H2 HA head-specific B cells from the CD19+ CD3/14/56 (dump)- CD27^{hi} CD38^{hi} CD20^{lo} CD21^{lo} plasmablast (A) or CD19+ CD3/14/56 (dump)- CD20+ IgG+ memory B cell compartment (B). Each plot shows the cell population gated immediately to the left as indicated above each plot. H2 HA head-specific B cells were detected as H2 HA ectodomain+ H2 stem-. (C) 2D classes of HA bound to polyclonal antibodies. Classes featuring pFabs with stem specificities outlined in red. (D) 3D reconstruction of stem-specific pFab.

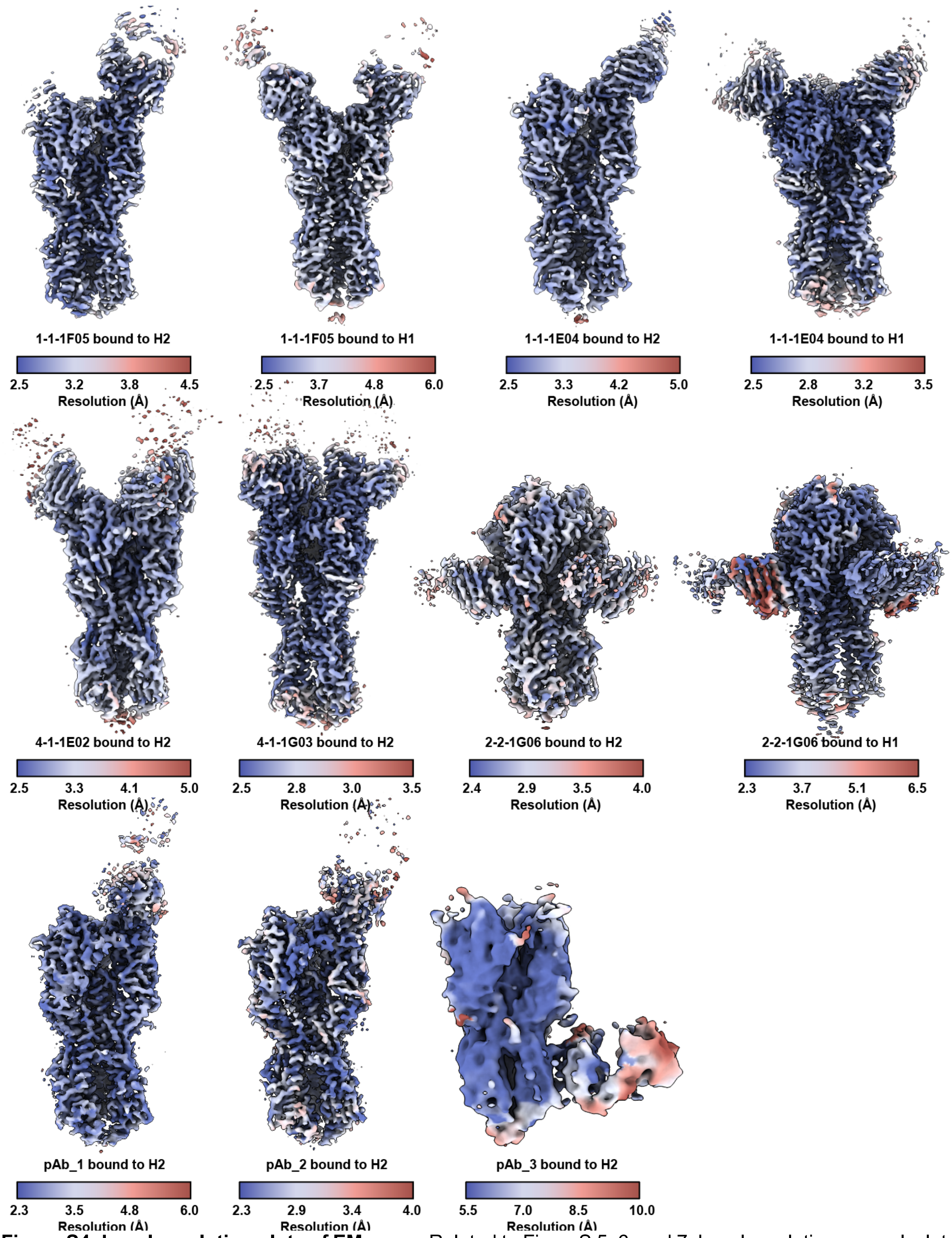

Figure S4: Local resolution plots of EM maps. Related to FigureS 5, 6, and 7. Local resolution was calculated according to a 0.143 FSC threshold in cryoSPARC 3.2 and visualized in ChimeraX.

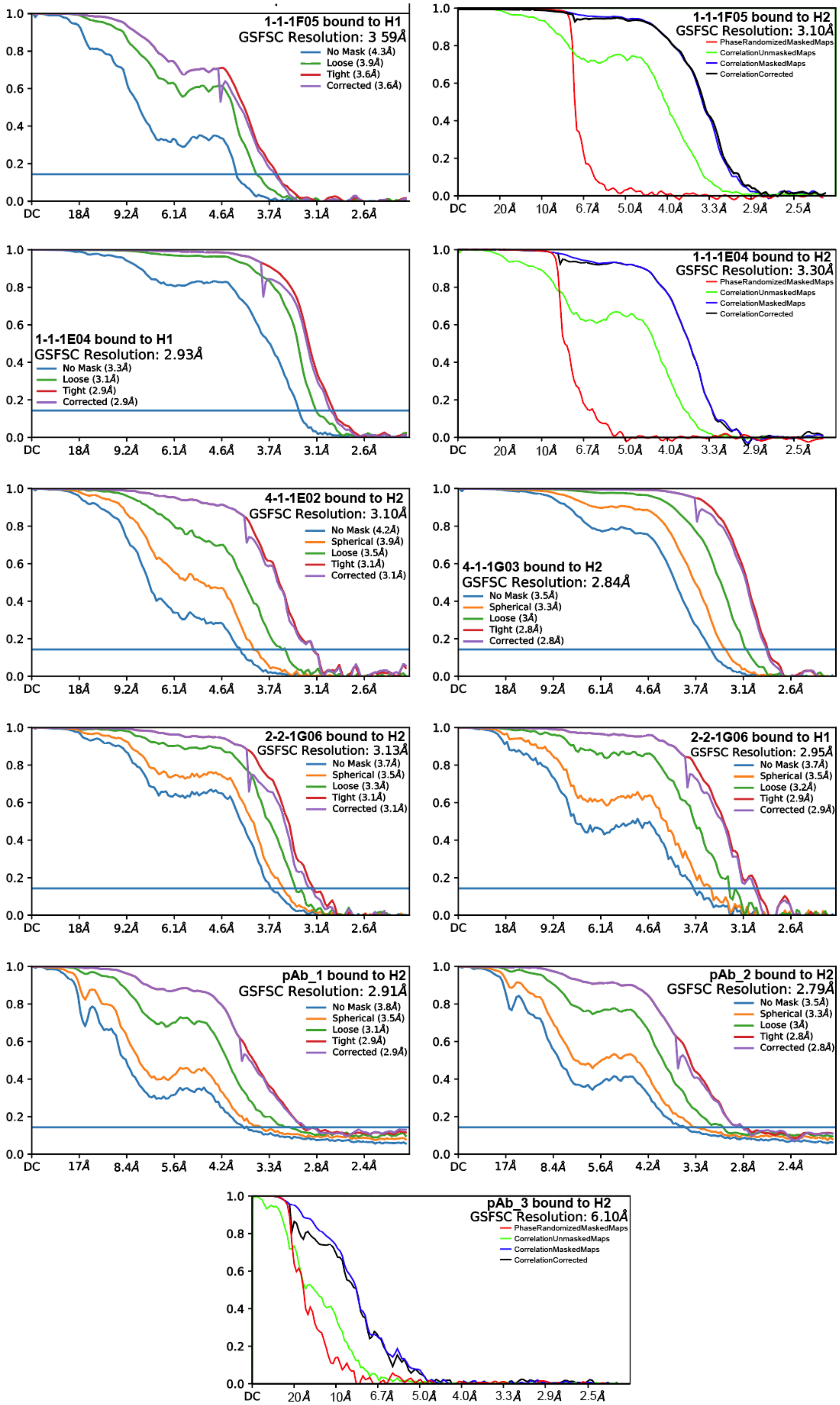

Figure S5: FSC plots for EM maps. Related to Figures 5, 6, and 7. Reported resolutions coincide with an FSC cutoff of 0.143. Plots were generated in cryoSPARC 3.2 or Relion.

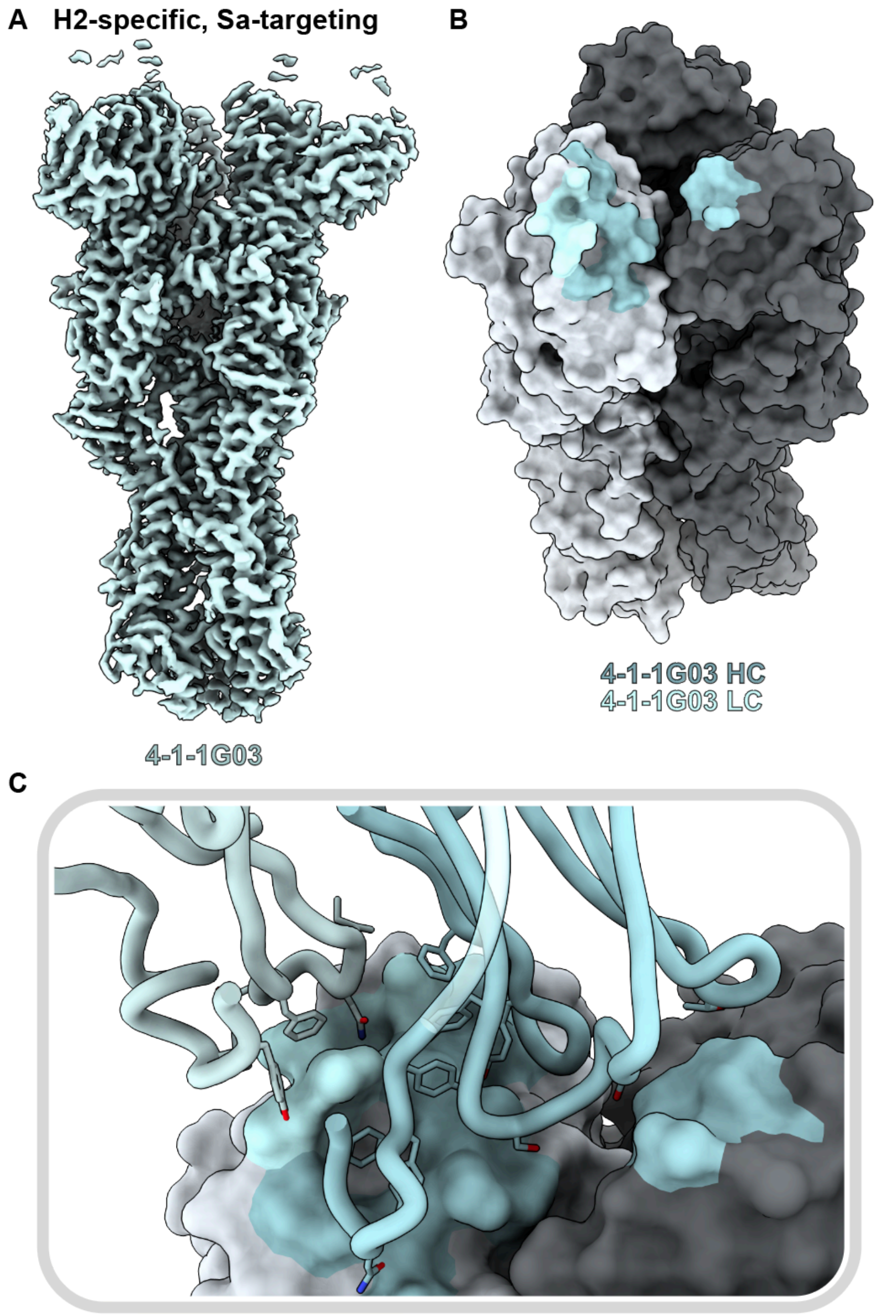

Figure S6: MAb 4-1-1G03 structural characterization. Related to Figure 6. (A) cryoEM density map of 4-1-1G03 Fab complexed with H2. (B) Antibody footprint of 4-1-1G03 colored to indicate heavy and light chain interactions on H2. (C) Antibody loop interactions with the SA epitope with key residues shown.

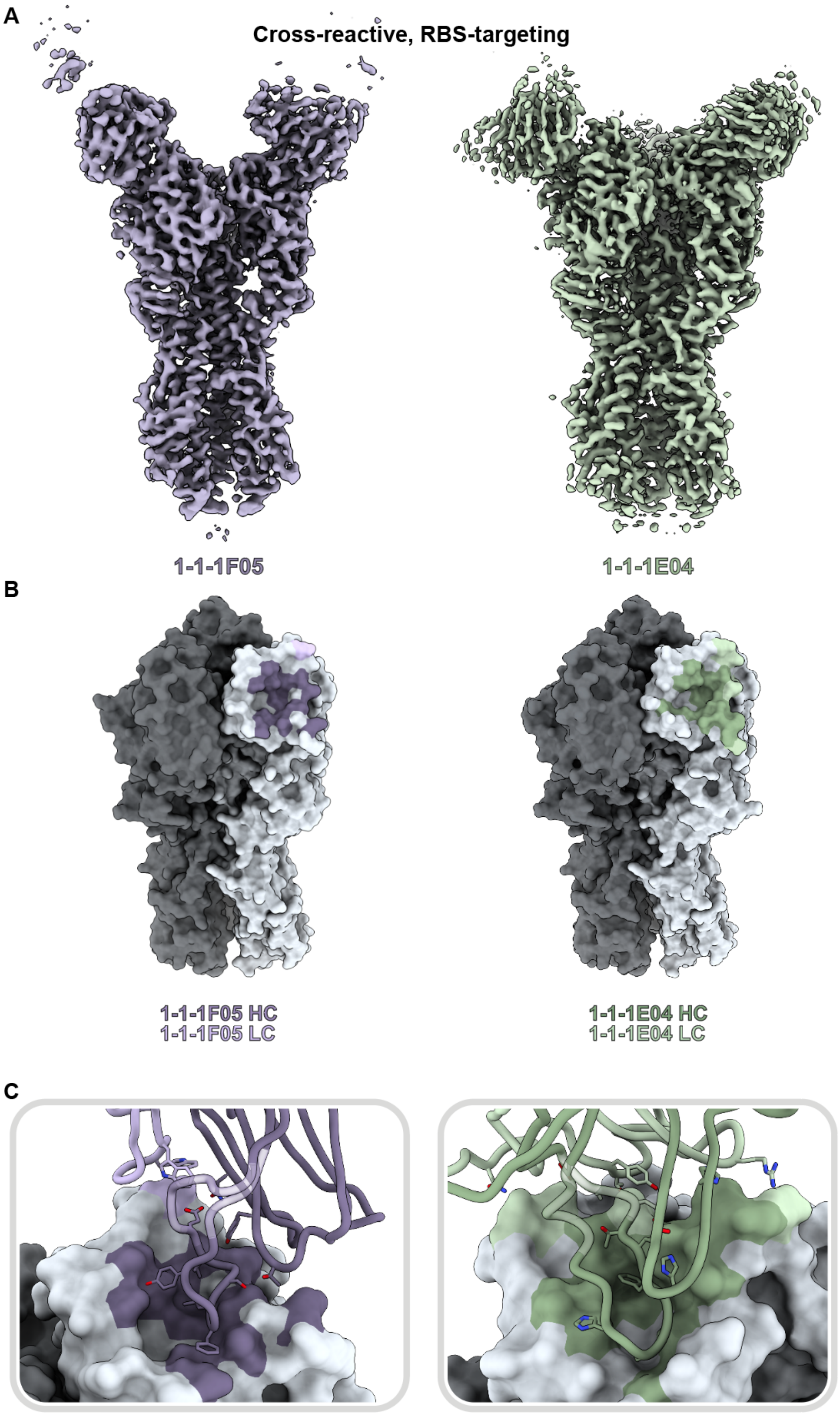

Figure S7: Cross-reactive mAb binding to H1 NC99. Related to Figure 6. (A) Cryo-EM reconstructions of mAbs 1-1-1F05 and 1-1-1E04 bound to H1 NC99. Antibody footprint of 1-1-1F05 and 1-1-1E04 colored to indicate heavy and light chain interactions on H1 NC99. (C) Antibody loop interactions with H1 NC99.

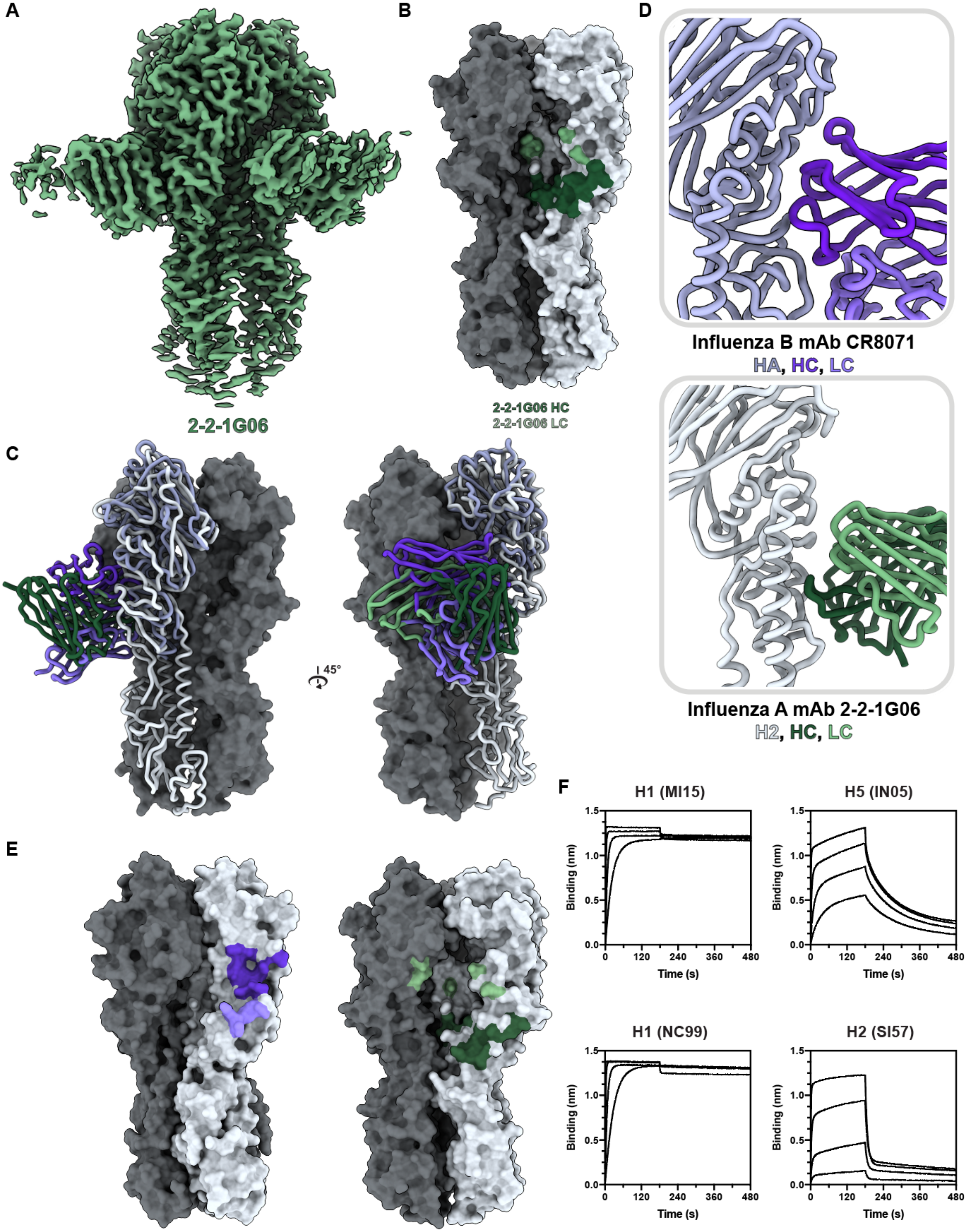

A
2-2-1G06
B
2-2-1G06 HC
2-2-1G06 LC
D
Influenza B mAb CR8071
HA, HC, LC
Influenza A mAb 2-2-1G06
H2, HC, LC
C
45°
E
F
H1 (MI15)
H5 (IN05)
H1 (NC99)
H2 (SI57)
Binding (nm)
Time (s)

Figure S8: MAb 2-2-1G06 interaction with H1 and comparison to CR8071 and binding kinetics. Related to Figure 7. (A) cryoEM density map of 2-2-1G06 Fab complexed with H1. (B) Antibody footprint of 2-2-1G06 colored to indicate heavy and light chain interactions on H1. (C & D) Comparison of influenza B mAb CR8071 (purple; PDB 4FQJ) bound to HA (PDBs 4FQJ and 4M44) and 2-2-1G06 (green) bound to H2. (E) Antibody footprints of 2-2-1G06 on H2 and CR8071 on HA (PDBs 4FQJ and 4FQM). (F) BLI sensorgrams indicating immobilized mAb 2-2-1G06 binding to H1, H2, or H5 HAs at concentrations of 3200, 800, 200, and 50 nM.

Table S1: nsEMPEM map and deposition details. Related to Figures 2 and 4.

	EMDB ID	Time point	# particles (composite)	Microscope	Pixel size
**Donor 1-1 (H2)**	EMD-41514	0	7,863	Tecnai TF20	1.77
	EMD-41515	4	11,200	Tecnai Spirit	2.06
	EMD-41516	16	17,496	Tecnai Spirit	2.06
	EMD-41517	20	51,147	Tecnai Spirit	2.06
**Donor 1-2 (H2)**	EMD-41518	0	2,167	Tecnai Spirit	2.06
	EMD-41519	4	9,223	Tecnai TF20	1.77
	EMD-41520	16	3,572	Tecnai Spirit	2.06
	EMD-41521	20	57,429	Tecnai Spirit	2.06
**Donor 1-3 (H2)**	EMD-41522	20	33,782	Tecnai Spirit	2.06
**Donor 2-1 (H2)**	EMD-41523	4	37,664	Tecnai TF20	1.77
	EMD-41524	16	80,050	Tecnai Spirit	2.06
	EMD-41525	20	48,917	Tecnai Spirit	2.06
**Donor 2-2 (H2)**	EMD-41526	0	5,252	Tecnai TF20	1.77
	EMD-41527	4	23,361	Tecnai Spirit	2.06
	EMD-41528	16	12,935	Tecnai Spirit	2.06
	EMD-41529	20	39,714	Tecnai Spirit	2.06
**Donor 2-3 (H2)**	EMD-41530	4	19,649	Tecnai Spirit	2.06
	EMD-41531	16	3,437	Tecnai TF20	1.77
	EMD-41532	20	16,270	Tecnai Spirit	2.06
**Donor 3-1 (H2)**	EMD-41533	0	7,280	Tecnai Spirit	2.06
	EMD-41534	4	27,785	Tecnai TF20	1.77
	EMD-41535	16	31,686	Tecnai Spirit	2.06
	EMD-41536	20	33,852	Tecnai Spirit	2.06
**Donor 3-2 (H2)**	EMD-41537	0	20,800	Tecnai TF20	1.77
	EMD-41538	4	19,649	Tecnai Spirit	2.06
	EMD-41539	16	41,400	Tecnai TF20	1.77
	EMD-41540	20	106,050	Tecnai Spirit	2.06
**Donor 3-3 (H2)**	EMD-41541	0	30,603	Tecnai Spirit	2.06
	EMD-41542	4	27,331	Tecnai Spirit	2.06
	EMD-41543	16	19,700	Tecnai Spirit	2.06
	EMD-41544	20	59,878	Tecnai Spirit	2.06
**Donor 4-1 (H2)**	EMD-41545	0	27,266	Tecnai Spirit	2.06
	EMD-41546	4	54,296	Tecnai Spirit	2.06
	EMD-41547	16	89,300	FEI Talos	1.98
	EMD-41548	20	93,574	Tecnai Spirit	2.06
**Donor 4-2 (H2)**	EMD-41549	0	18,505	Tecnai Spirit	2.06
	EMD-41550	4	50,289	Tecnai Spirit	2.06
	EMD-41551	16	10,340	Tecnai Spirit	2.06
	EMD-41552	20	49,813	Tecnai TF20	1.77
**Donor 4-3 (H2)**	EMD-41553	0	11,809	Tecnai Spirit	2.06
	EMD-41554	4	82,814	Tecnai Spirit	2.06
	EMD-41555	16	14,356	Tecnai Spirit	2.06
	EMD-41556	20	66,790	FEI Talos	1.98
**Donor 1-1 (H1)**	EMD-41557	0	33,500	Tecnai TF20	1.77
	EMD-41558	4	26,600	Tecnai TF20	1.77
	EMD-41559	16	19,300	Tecnai TF20	1.77
	EMD-41560	20	53,500	Tecnai TF20	1.77
**Donor 2-2 (H1)**	EMD-41561	0	34,000	Tecnai TF20	1.77
	EMD-41562	4	88,000	Tecnai TF20	1.77
	EMD-41563	16	65,400	Tecnai TF20	1.77
	EMD-41564	20	45,700	Tecnai TF20	1.77

Table S2: Cryo-EM map and atomic model refinement. Related to Figures 5, 6, and 7.

	**1-1-1F05 bound to H2**	**1-1-1F05 bound to H1**	**1-1-1E04 bound to H2**	**1-1-1E04 bound to H1**	**4-1-1E02 bound to H2**	**4-1-1G03 bound to H2**	**2-2-1G06 bound to H2**	**2-2-1G06 bound to H1**	**pAb_1 bound to H2**	**pAb_2 bound to H2**	**pAb_3 bound to H2**
**Access codes**											
PDB	8TP2	8TP3	8TP4	8TP5	8TP6	8TP7	8TP9	8TPA	N/A	N/A	N/A
EMDB	EMD-41464	EMD-41465	EMD-41466	EMD-41467	EMD-41468	EMD-41469	EMD-41470	EMD-41471	EMD-41472	EMD-41473	EMD-41474
GenBank	BAF48641.1	AAP34324.1	BAF48641.1	AAP34324.1	BAF48641.1	BAF48641.1	BAF48641.1	AAP34324.1	N/A	N/A	N/A
**Data collection and processing**											
Microscope	Talos Arctica	Talos Arctica	Talos Arctica	Talos Arctica	Talos Arctica	Talos Arctica	Talos Arctica	Talos Arctica	Titan Krios	Titan Krios	Talos Arctica
Magnification	36,000	36,000	36,000	36,000	36,000	36,000	36,000	36,000	130,000	130,000	36,000
Voltage (kV)	200	200	200	200	200	200	200	200	300	300	200
Electron exposure ($e^-/Å^2$)	53.5	49.0	49.2	46.2	49.0	49.0	46.2	48.6	49.7	49.7	50.3
Defocus range (μm)	-0.7 to -2	-0.7 to -2	-0.7 to -2	-0.7 to -2	-0.7 to -2	-0.7 to -2	-0.7 to -2	-0.7 to -2	-0.7 to -2	-0.7 to -2	-0.7 to -2
Pixel size (Å)	1.150	1.15	1.150	1.15	1.150	1.150	1.150	1.150	1.045	1.045	1.045
Imposed Symmetry	C1	C1	C1	C3	C3	C3	C3	C3	C1	C1	C1
Final particle number	230,649	105,408	117,851	167,166	103,798	271,581	164,150	124,412	39,631	61,108	17,933
Map resolution (Å)	3.1	3.6	3.3	2.9	3.1	2.8	3.1	3.0	2.9	2.8	6.1
FSC Threshold	0.143	0.143	0.143	0.143	0.143	0.143	0.143	0.143	0.143	0.143	0.143
Map sharpening B-factor ($Å^2$)	-37.9	-99.7	-40.8	-95.0	-94.9	-105.9	-99.6	-83.7	-56.3	-58.8	-138.4
**Model refinement and validation**											
Total Residues	1697	1720	1697	2223	2153	2163	2157	2172			
Amino-acids	1678	1700	1678	2205	2126	2145	2142	2154			
Carbohydrates	19	20	18	18	27	18	15	18			
RMSD Lengths (Å)	0.020	0.020	0.020	0.021	0.021	0.021	0.020	0.021			
RMSD Angles (°)	1.7	1.7	1.8	1.9	1.8	1.8	1.8	1.8			
**Ramachandran**											
Outliers (%)	0	0	0	0	0	0	0	0			
Allowed (%)	2.2	1.7	2.7	1.8	1.2	1.7	1.9	1.3	N/A	N/A	N/A
Favored (%)	97.8	98.28	97.4	98.21	98.8	98.3	98.1	98.7			
Rotamer outliers (%)	0	0.07	0	0.11	0	0	0	0			
Clash score	1.6	0.8	2.9	1.6	1.9	1.4	2.3	1.5			
Molprobity score	0.94	0.75	1.21	0.91	0.95	0.88	1.00	0.89			
FSC model (0/0.143/0.5)	2.7/2.8/3.1	3.5/3.5/3.9	2.9/3.0/3.3	2.7/2.8/3.1	3.0/3.1/3.3	2.8/2.8/3.0	3.0/3.1/3.3	2.9/2.9/3.2			
EMRinger score	4.2	2.4	4.0	3.4	3.7	4.8	3.5	4.7			

Table S3: nsEM map and deposition details for monoclonal immune complexes. Related to Figures 4 and 5.

Monoclonal ID	EMDB ID	Breadth	# particles (composite)	Symmetry	Microscope	Pixel size (Å)
**2-2-1E08**	EMD-41683	H2	3,672	C1	Tecnai Spirit	2.06
**2-2-1C06**	EMD-41684	H2	11,091	C1	Tecnai Spirit	2.06
**4-1-1E02**	EMD-41685	H2	25,927	C1	Tecnai Spirit	2.06
**4-1-1G03**	EMD-41686	H2	14,494	C3	Tecnai Spirit	2.06
**1-3-1F08**	EMD-41687	H2	4,424	C1	Tecnai Spirit	2.06
**1-1-1F05**	EMD-41688	cross-reactive	2,741	C1	Tecnai Spirit	2.06
**2-2-1F01**	EMD-41689	cross-reactive	22,641	C1	Tecnai Spirit	2.06
**2-2-1G06**	EMD-41690	cross-reactive	2,651	C1	Tecnai Spirit	2.06
**1-2-189-34**	EMD-41691	cross-reactive	29,435	C3	Tecnai F20	1.77
**1-1-1A09**	EMD-41692	cross-reactive	17020.00	C3	Tecnai F20	1.77
**1-1-2A11**	EMD-41693	cross-reactive	4,231	C3	Tecnai F20	1.77
**1-1-2E05**	EMD-41694	cross-reactive	8,762	C3	Tecnai F20	1.77
